# Non-invasive Neurite Mechanics in Differentiated PC12 Cells

**DOI:** 10.3389/fncel.2018.00194

**Published:** 2018-07-06

**Authors:** Fernanda Gárate, María Pertusa, Yahaira Arana, Roberto Bernal

**Affiliations:** ^1^Cellular Mechanics Laboratory, Physics Department, SMAT-C, University of Santiago, Santiago, Chile; ^2^Biophysics Laboratory, Physics Department, SMAT-C, University of Santiago, Santiago, Chile; ^3^Department of Biology, Millennium Nucleus of Ion Channels-Associated Diseases (MiNICAD), University of Santiago de Chile, Santiago, Chile

**Keywords:** neurite mechanics, cytoskeleton, plasma membrane, noise spectroscopy, novel instrumentation

## Abstract

Thermal Fluctuations Spectroscopy (TFS) in combination with novel optical-based instrumentation was used to study mechanical properties of cell-cultured neurites with a spatial resolution limited only by the light diffraction. The analysis of thermal fluctuations together with a physical model of cellular elasticity allow us to determine relevant mechanical properties of neurite as axial tension σ, flexural rigidity B, plasma membrane tension γ, membrane bending rigidity K, and cytoskeleton to membrane-coupling ρ_*b*_*k*, whose values are consistent with previously reported values measured using invasive approaches. The value obtained for the membrane-coupling parameter was used to estimate the average number of coupling elements between the plasma membrane and the cytoskeleton that fell in the range of 30 elements per area of the laser spot used to record the fluctuations. Furthermore, to expand the TFS analysis, we investigate the correlation between F-actin linear density and the mechanical features of PC12 neurites. Using a hybrid instrument that combines TFS and a simple fluorescent technique, our results show that the fluctuations are related with the F-actin concentration. These measurements have an advantage of not requiring the application of an external force, allowing as to directly establish a correlation between changes in the mechanical parameters and cytoskeleton-protein concentrations. The sensibility of our method was also tested by the application of TFS technique to PC12 neurite under Paraformaldehyde and Latrunculin-A effect. These results show a dramatic modification in the fluctuations that are consistent with the reported effect of these drugs, confirming the high sensitivity of this technique. Finally, the thermal fluctuation approach was applied to DRG axons to show that its utility is not limited to studies of PC12 neurites, but it is suitable to measure the general characteristic of various neuron-like cells.

## 1. Introduction

The Thermal Fluctuations Spectroscopy (TFS) has been used in a variety of biological systems, allowing one to obtain mechanical information about such systems with a high temporal and spatial resolution in a noninvasive way. There is a strong evidence that this technique can be successfully applied using traditional optical tweezers setups (Betz et al., 2009; Peukes and Betz, 2014; Gárate et al., 2015). In order to accurately interpret the data, the TFS measurements must be complemented with an underlying physical model of the system. Therefore, the more complete the theoretical mechanical model is, the more accurate will be the assessment of the mechanical parameters.

Aiming to give to non-physicist readers a general overview of the experimental technique, the mathematical framework, the biological scales that are involved, the experimental considerations and the limitations of the technique, we will introduce a brief theoretical description of the optical tweezers and the physical models that have been developed to obtain mechanical information of neuron-like cells using TFS measurements.

### 1.1. Using optical tweezers for TFS measurements

The optical tweezers (OT) are easy to implement in traditional inverted microscopes and are extremely versatile in lab-made modular configurations (Ashkin, 1997; Neuman and Block, 2004). In traditional OT experiments, a dielectric micron-size bead is usually used to apply forces (Moffitt et al., 2008). Moreover, OT technique can be used to sense forces by the detection of the trapped bead position relative to the center of the laser spot (trap), whose image is projected on a photodiode quadrant detector (Gittes and Schmidt, 1998; Dreyer et al., 2004). Close to the equilibrium position, the thermal movement of the trapped bead can be modeled as a damped particle-spring system with an off-center displacement within 1μm range, that can be described by the Langevin's equation (Kampen, 1992). Using the fluctuation-dissipation theorem (Evans and Searles, 2002; Marconi et al., 2008), it is possible to quantify the fluctuations of the trapped bead and relate these fluctuations to the physical characteristics of the medium.

Another technical aspect of the OT systems is the trapping force exerted over the bead, which is proportional to the laser power and the size of the trapped bead. Therefore, the OT can be easily switched between a stiff trap and a soft trap. This property makes the OT a useful tool that can be applied to a wide range of biological systems. The versatility of OT systems to tune the restitution force has been widely exploited to measure the DNA stretching elasticity (Wang et al., 1997; Bennink et al., 1999; Bockelmann et al., 2002), the power stroke and stepping size of molecular motors (Block et al., 1990; Howard, 1996; Veigel et al., 1999; Tyska and Warshaw, 2002), the surface tension of the plasma membrane (Dai and Sheetz, 1999; Betz and Sykes, 2012; Peukes and Betz, 2014), the mechanical response of artificial F-actin bundles (Rückerl et al., 2017). However, all these experiments have been interpreted using a force-displacement curve that takes significant time to obtain.

One way to obtain mechanical information, in a shorter time, is by using the TFS technique. This technique records the fluctuations of a biological sample, taking advantage of the difference between the refractive index of the biological sample and the medium. If the laser is located at the edge of the biological sample, its light path will be deflected according to the fluctuations of the sample edge, allowing a direct analysis of these fluctuations in the Fourier space for the computation of the Power Spectrum Density of the signal (*PSD*). In order to convert the recorded signal into nano-metric fluctuations of the sample, a calibration curve is needed, which can be easily obtained by recording the deflection of the laser while moving the sample edge in a controlled way (Capitanio et al., 2002; Gögler et al., 2007; Jun et al., 2014). This strategy has been successfully used to obtain mechanical information about vesicle lipid membranes (Betz and Sykes, 2012; Lemière et al., 2013), red blood cells (Betz et al., 2009; Turlier et al., 2016), cell motility through membrane blebbing (Peukes and Betz, 2014) and in PC12 neurites (Gárate et al., 2015).

### 1.2. Thermal fluctuations of a membrane and a string

In order to extract mechanical information from fluctuation-based measurements, it is necessary to record the transversal amplitude of the fluctuation as a function of time denoted by *h*(*t*) at a given location of the sample. Thus, the analysis of the signal *h*(*t*) will allow us to compute the Power Spectral Density (*PSD*) which is subsequently compared to the theoretical *PSD* of the system. From this comparison, the mechanical parameters are obtained.

**Figure 1:**
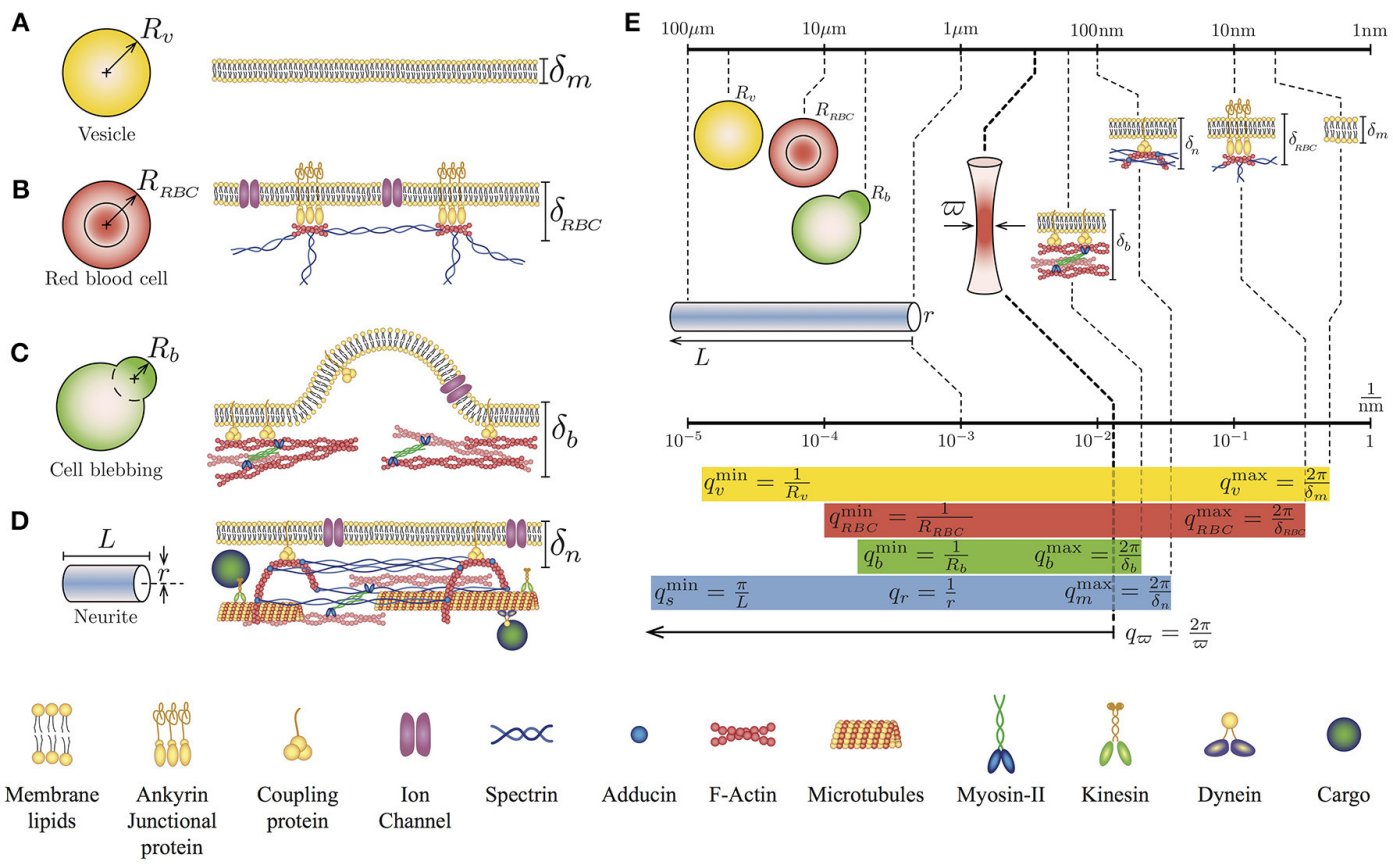
Geometry and simplified cytoskeletal architecture of **(A)** Giant Unilamellar Vesicle of radius *R*_*v*_ and lipid membrane thickness δ_*m*_, **(B)** Human red blood cell of radius *R*_*RBC*_ and membrane thickness δ_*RBC*_, **(C)** Bleb of a blebbing-based motility cell *R*_*b*_ and membrane thickness δ_*b*_, and **(D)** Neurite of length *L* radius *r* and membrane thickness δ_*n*_. Note that the membrane thickness δ is referred to the plasma membrane-cytoskeleton thickness. **(E)** Comparison of the lengths scales and *q* wave-vectors for the different cell types and cytoskeletal architecture in relation to the waist size of the laser spot ϖ. The *q* values for each case correspond to the theoretical integration limits of Equations (1) and (2).

One of the first theoretical expression of the *PSD* for a biological system describes the membrane fluctuations of vesicle-like objects (Betz et al., 2009; Betz and Sykes, 2012) (Figures 1A–C). This theoretical expression (Equation 1) considers the bending rigidity K (Dimova, 2014; Simunovic et al., 2015) and the surface tension γ (Hochmuth, 2000; Tinevez et al., 2009) of the plasma membrane. The model also considers the coupling between the plasma membrane and the elastic inner cortex ρ_*b*_*k* (Alert et al., 2015), where ρ_*b*_ is the density of available linkers and *k* is the linker stiffness. Furthermore, the dynamical viscosity η takes into account the effect of the surrounding liquid media. However, due to the viscosity of the inner fluid of a vesicle-like object, which can be different from the external viscosity, it is convenient to define the effective viscosity $\eta_{m}^{e}$, which accounts for the external and the cytosol viscosity separated by the plasma membrane (Supplementary Material of reference Betz and Sykes, 2012).

(1) $$\begin{array}{l} PSD_{m}=\frac{4\eta_{m}^{e}k_{B}T}{\pi }\int_{q_{m}^{\min}}^{q_{m}^{\max}}\frac{dq}{{\left(Kq^{3}+\gamma q+\rho_{b}k/q\right)}^{2}+{\left(4\eta_{m}^{e}\omega \right)}^{2}} \end{array}$$

This description relates the normal modes to the frequency of oscillation of a flat membrane close to a rigid substrate (Prost et al., 1998). Although vesicles are not flat, the *PSD*_*m*_ of a flat membrane is not far from a spherical membrane (Betz and Sykes, 2012; Alert et al., 2015). Then, the main advantage of the flat case is the model simplicity.

This theoretical approach has been used to investigate the mechanical properties of vesicles (Betz and Sykes, 2012), the ATP dependency of red blood cells mechanical properties (Betz et al., 2009), and the cortical tension on membrane blebs (Peukes and Betz, 2014). Notice that the integration limits of the Equation (1) are defined by the geometrical features of the sample, for instance $q_{m}^{\min}=1/R$ and $q_{m}^{\max}=2\pi /\delta$, where *R* is the radius of the vesicle-like object and δ is the membrane thickness (Figures 1A–C). Although the solution of Equation 1 has no analytical form, it can be computed approximately. Using the membrane dispersion relation, it is possible to establish the range of frequencies where the solution is valid (Supplementary Material of reference Betz et al., 2009).

The TFS technique can be also extended to include more particular cellular architectures (Figure 1D). Indeed, we have recently demonstrated, both experimental and theoretically, that PC12 neurites exhibit in addition to thermal-driven fluctuations of their plasma membrane, other fluctuations coming from their string-like geometry (Gárate et al., 2015). Here, the theoretical *PSD* considers the tension along the neurite σ (Lamoureux et al., 1989) and the neurite flexural rigidity B (Gittes et al., 1993; Kikumoto et al., 2006). In terms of dissipation, the effective viscosity $\eta_{s}^{e}$ is composed by the external viscosity of the medium (Supplementary Material of reference Gárate et al., 2015), where the theoretical expression for the string-like geometry is:

(2) $$\begin{array}{l} PSD_{s}=\frac{\eta_{s}^{e}k_{B}T}{\pi }\int_{q_{s}^{\min}}^{q_{s}^{\max}}\frac{dq}{{\left(Bq^{4}+\sigma q^{2}\right)}^{2}+{\left(\eta_{s}^{e}\omega \right)}^{2}} \end{array}$$

A major advantage of working with neuron-like cells is an extra length-scale found in their geometry. For instance, in round cells, we could distinguish two length scales: the thickness δ of the plasma membrane and the radius of both the cell and the cytoskeletal cortex, defined by *R*. In contrast, in the neurite case, we can identify the thickness of the plasma membrane, the length *L* of the neurite and its radius *r* (Figure 1). Therefore, the amplitude of the fluctuations comes from two sources: from the membrane and the string, that can be written as: *h*(*x, t*) = *h*_*m*_(*x, t*) + *h*_*s*_(*x, t*). Taking into account that for a neurite, the associated *q*-vectors are linked to different geometries and different dispersion relationships (membrane and string), the cross-correlations between *h*_*m*_ and *h*_*s*_ can be neglect (Gárate et al., 2015). Then, the temporal correlation of the fluctuation amplitude *h*(*t*) is given by 〈*h*(*t*)*h*(0)〉 = 〈*h*_*m*_(*t*)*h*_*m*_(0)〉 + 〈*h*_*s*_(*t*)*h*_*s*_(0)〉. Thus, the theoretical expression for the complete *PSD* of neuron-like cells *PSD*_*n*_ is the arithmetic sum of the two previous results (Equations 1 and 2).

(3) $$\begin{array}{l} PSD_{n}=PSD_{m}+PSD_{s} \end{array}$$

We need to point out that, this last result (Equation 3) rules out the existence of a power-law *f*^−2/3^ in the neurite power spectrum which was proposed in reference (Gárate et al., 2015).

The integration limits for the first term of Equation (3) are: $q_{m}^{\min}=q_{r}=1/r$ and $q_{m}^{\max}=2\pi /\delta_{n}$. Whereas, the limits for the second term are: $q_{s}^{\min}=\pi /L$ and $q_{s}^{\max}=1/r$ (Figure 1E). Furthermore, the membrane and the string fluctuations are damped by the cytosol-extracellular fluid and cytoskeleton-extracellular fluid, whose effective viscosities are $\eta_{m}^{e}$ (Betz et al., 2009; Betz and Sykes, 2012) and $\eta_{s}^{e}$ (Hill et al., 2004; Gárate et al., 2015). With this description and the TFS measurements it is possible to assess, simultaneously, the key mechanical features of both the plasma membrane and the cytoskeleton of neuron-like cells.

Our results show that the TFS technique is sensitive enough to distinguish between mechanical parameters of PC12 neurites of apparently similar radius and length. Furthermore, by incorporating fluorescence imaging, we have established a positive correlation between the linear density of F-actin and the axial tension in PC12 neurites. Moreover, the TFS measurements show the dramatic changes of the mechanical features when the neurites are treated with Latrunculin-A (Lat-A) and Paraformaldehyde (PFA). Finally, we show that this technique can also be used to study axons from primary sensory neurons in culture.

## 2. Materials and methods

### 2.1. Experimental setup

**Figure 2:**
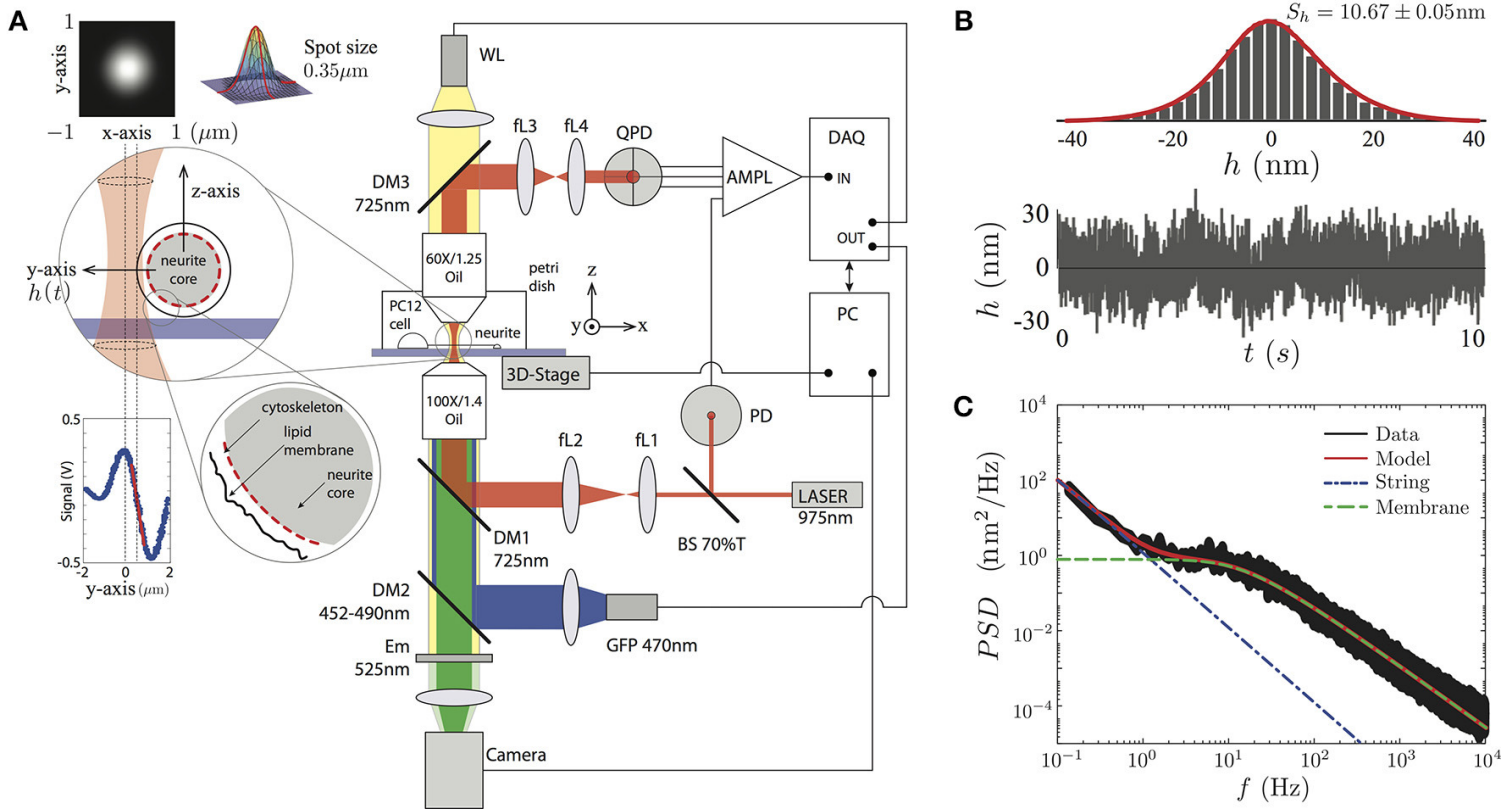
**(A)** Sketch of the experimental setup. The PC12 neurites are imaged by a white lamp (WL) placed at the top of the setup or by the fluorescent lamp (LED) placed after the IR laser. The PC12 neurite edge is placed near the highly focused laser (less than 10mW). The light scattered by the neurite edge is imaged on a QPD sensor, amplified and recorded by the DAQ card. The conversion from voltages to displacements at the QPD sensor is performed using the slope of the signal in the linear region (red segment at the Signal vs. Position inset plot). The whole system is supported by a passive vibration-damping optical table. **(B)** The temporal transverse amplitude fluctuation, *h*(*t*), is acquired during 10 s, displays a Gaussian probability amplitude distribution of a particular PC12 neurite. **(C)** The *PSD* is obtained over four decades in the frequency space. The full model (red continuous line), the string (blue point-segmented line), and the membrane (green segmented line) models are shown to be in a complete agreement with the experimental data.

To record the transverse amplitude fluctuations of PC12 neurites, in the plane and perpendicular to the neurite long axis (Figure 2A), we built an optical tweezers by using an infrared laser (PL980P330J 330 mW power and λ = 975 nm, Thorlabs) whose power is set to less than 10mW in the sample plane (Figure 2). The output laser power is maintained by a current and temperature PID controller (LDC210C and TED200C, Thorlabs) and monitored by a photodiode sensor (PD) (DET10N, Thorlabs). The resulting beam is imaged on the back focal plane of an oil immersion objective (Olympus 100 × /1.4 Oil) by a ten-fold magnification telescope (with focal distances of the lenses being fL1 = 20 mm, fL2 = 200 mm). The light is coupled in the optical path by a dichroic mirror (DM1). After interacting with the neurite, the light ray is collected by an objective (Olympus 60 × /1.25 Oil) used as a condenser. The light is imaged on the back focal plane of a quadrant photodiode (QDP) (PDQ80A, Thorlabs) via a second telescopic system, composed of two lenses (fL3 = 50 mm, fL4 = 25.4 mm). The QPD signal is processed by a low-noise amplifier and acquired by a data acquisition card (NI PCI-6251, National Instruments, Austin, TX). The instrument control, data recording, and image acquisition are synchronized by LabView control software (National Instruments).

To visualize the PC12 cell sample, we use the WL lamp (MCWHL2 LED, Thorlabs) placed at the top of the setup (Figure 2A), from which a bright field image is formed at the camera port of the setup (DCC3240N, Thorlabs) and recorded by the computer. The setup also incorporates a second high power diode lamp for GFP fluorescent imaging (M470L2 blue LED, Thorlabs) coupled to the optical path through a second dichroic mirror (DM2, 452–490 nm) and an emission filter (Em, 525 nm) is placed in front of the camera.

In order to get access only to the neurite amplitude fluctuations, and not to other cellular processes, the TFS measurements were performed at the middle of the neurite shaft. Then, we perform the calibration for each sample by scanning laterally the edge of the neurite as shown in Figure 2A. This procedure is repeated 10 times in order to reduce the statistical error of the conversion factor in the linear regime (the conversion factor relative error was (5.57 ± 1.06)% among all the PC12 neurites) between the laser spot position and the voltage of the QPD sensor (Figure 2A, inset panel). The data is then analyzed using Matlab software (The MathWorks, Natick, MA) in order to compute mean values and power spectral densities (using the FFT algorithm). The mechanical parameters are obtained by fitting the experimental data to the Equation (3).

### 2.2. Experimental limitations

The different biological systems displayed in the Figure 1 are described in terms of their geometrical features and their membrane-cortex composition. Therefore, to access only the mechanical parameters of the cytoskeleton and the plasma membrane of the neurite, the experiments were carried out approximately in the middle of the neurite shaft. Near the soma or the neurite growth cone, the TFS technique could only access the fluctuations of the membrane due to the strong adhesion of these regions to the substrate.

Each neurite feature contributes to a range of *q*-modes that will be integrated in order to measure, for a single point of the sample, the temporal amplitude of these fluctuations. Experimentally, the available *q*-modes are limited by the finite spot size of the laser ϖ = λ/2NA ≈ 350 nm, implying that the high limit integration in the theoretical expression of the *PSD*_*n*_ will be *q*_ϖ_ = 2π/ϖ, with λ as the laser wavelength and NA as the numerical aperture of the microscope objective used to focus the laser. As shown in Figure 1E, the size of the spot of the laser, characterized by ϖ, is at least 30 times the size of the lipid membrane thickness δ_*m*_ ≈ 10 nm. Nevertheless, this limitation does not imply that we are not able to detect the contribution of features below to the value of ϖ. In fact, due to the dispersion relationships, each geometrical feature in combination with the mechanical properties of the sample can be observed in a range of frequencies in the Fourier spectrum (Figure 1E).

In the frequency domain, the range is limited by the acquisition time (typically set to 10 s), leading to a lower frequency cutoff 0.1 Hz. For longer time scales, the thermal drift of the instruments limits the detection of nanometrically-precise fluctuations. At high frequencies, the limit of the measurement is set by three main factors: the signal acquisition rate (200 kHz), the bandwidth of the quadrant photodiode detector (150 kHz) and the background noise of the whole experimental apparatus, resulting in a upper frequency limit of the order of 10 kHz at which the detection limit is of the order of 10^−5^nm^2^/Hz.

### 2.3. Laser power considerations

In contrast to RBC, the amplitude of the PC12 neurites fluctuations does not depend on the applied laser power in a range of 2–200 mW, which at the focal plane is reduced to a 0.5–50 mW (Supplementary Data of reference Gárate et al., 2015). This difference is explained by two factors: the poor cytoskeleton and the rich hemoglobin environment in RBC vs. a complex cytoskeleton and the small index refraction difference of the neurites. However, the heat produced by a highly focused laser could potentially modify the mechanical properties and even damage the neurite. This heat is proportional to the laser power, therefore the optical tweezers are operated at the power within a certain range to avoid mechanical disturbance. An estimated value for the increase in temperature of the sample caused by the laser is the following. The power absorption by the sample is given by *P*_*abs*_ = Δ*T C*_*p*_ *m*/τ, the transmittance of the sample $𝕋=e^{-\mu_{\lambda }\ell }$, *P*_*abs*_ = (1 − *𝕋*)*P*_*o*_, and the thermal diffusion in the sample $\partial_{t}T=\alpha \nabla^{2}T$ where *T* is the temperature, *C*_*p*_ = 4.184 J·g^−1^·K^−1^ is the water heat capacity, *m* = ρ_*water*_*v* is the mass of water in the volume $v\approx \varpi^{2}\cdot \varpi_{z}\approx (0.35\mu$m)^2^(0.78μm) with ϖ_*z*_ ≈ πλ/2NA, τ is the characteristic time to equilibrate a temperature change, μ_λ_ = 10 m^−1^ is the water optical absorption coefficient at λ = 975 nm, ℓ ≈ ϖ_*z*_ is the optical length, and α = 0.143 × 10^−6^ m^2^/s is the water thermal diffusion coefficient. Then, from the thermal diffusion equation, the characteristic time of the system is obtained from Δ*T*/τ ≈ αΔ*T*/ℓ^2^ → τ = ℓ^2^/α ≈ 4.3 × 10^−6^ s. Using the above relationships and setting the laser power at *P*_*o*_ = 10 mW, results in a temperature increase in the plane of the sample of about two degrees. Thus, the laser power set at 10 mW is a reasonable limit to measure neurite fluctuations without temperature effects over the mechanical parameters (Spedden et al., 2013). Moreover, operating the laser at powers below 2 mW reduces the spectrum window to approximately 1kHz due to the signal to noise ratio for higher frequencies (Supplementary Data of reference Gárate et al., 2015).

### 2.4. Cell culture, F-actin labeling, and quantification

Wild-type PC12 cells (CRL-1721, www.atcc.org) were cultivated in media DMEM (Gibco 12430) contained 6% of Horse Serum (Gibco 16050), 5% of Fetal Bovine Serum (Gibco 10437) and 1% of antibiotic-antimycotic complement (Gibco 15240). For experiments, modified coverglass bottom Petri dishes are used. The cover glass is coated with collagen solution at a 50μg/mL (Gibco A1064401) and the cells are seeded at a concentration of 10^4^ cells/mL. In this step, the cells are cultivated for 4 days in a modified culture media by adding 30 ng/mL of nerve growth factor NGF-2.5 (Gibco 13257). Under these conditions, isolated PC12 neurites of considerable length are obtained (100–200 μm).

We generate a stable PC12 cell line expressing the mEGFP-Lifeact-7 construct (www.addgene.org #54610). The transfection of the plasmid was performed using the standard Lipofectamine 2000 protocol (Invitrogen 11668019). The stable PC12 cell line expressing mEGFP-Lifeact was obtained using G418 as a selection marker.

**Figure 3:**
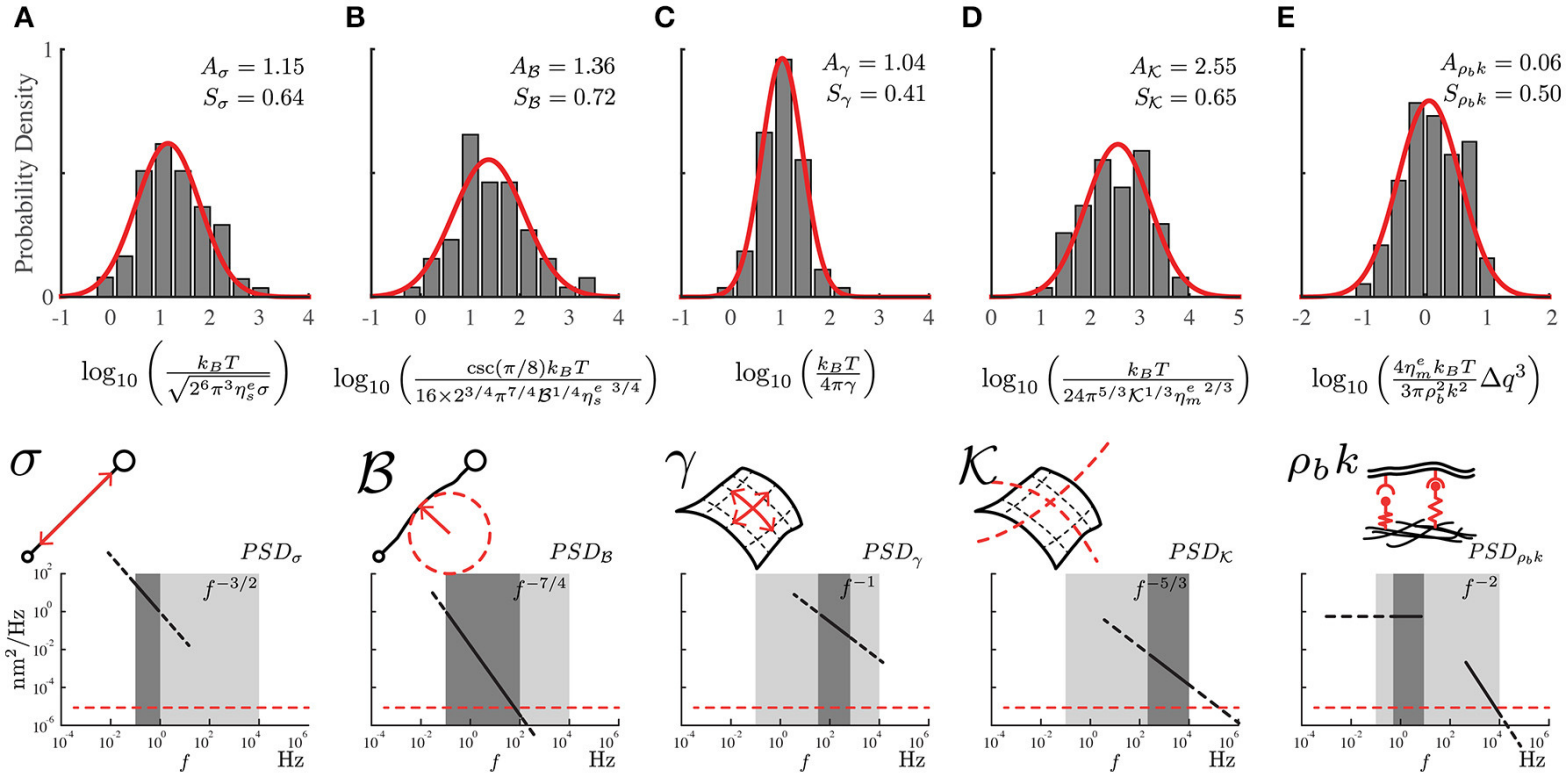
The probability distribution of the mechanical properties for the PC12 cell neurites using the local power-laws and amplitudes for each frequency regime. **(A)** Axial tension σ, **(B)** Flexural rigidity B. **(C)** Plasma membrane surface tension γ. **(D)** Plasma membrane bending K and **(E)** Membrane to cytoskeleton coupling ρ_*b*_*k*. The mechanical parameters are represented in the sketches including the approximate range of frequencies and the theoretical power-laws for each one of them (dark gray region). The light gray region represents the range of available frequencies while the red segmented line represents the low detection limit of the instrument. The samples were prepared weekly, we recorded four to six neurites per week reaching a total of 75 neurites (*N* = 75).

LifeAct is a fusion protein consisting of 17 amino acids from the Actin Binding Protein-120 (ABP-120) with GFP that offers us the opportunity to visualize actin in living cells (Riedl et al., 2008; Normoyle and Brieher, 2012). We quantified the Lifeact fluorescence prior to the TFS measurement. The neurite fluorescent images were acquired using 200 ms of exposition with zero gain in all experiments. Then, the linear F-actin density was computed as ρ_*l*_ = Integrated intensity/neurite segment. The maximum linear density ρ_*o*_ was assumed to be equal to the saturation intensity of the camera of a neurite of radius 1.18μm (Figure 3C).

The Dorsal Root Ganglion cells (DRG) were extracted from young adult P21 C57BL6 mice and incubated in an enzymatic mixture including collagenase type XI (650 UI/mL; Sigma C7657) and dispase (5 UI/mL; GIBCO 17105041), in INC-mix solution (in mM: NaCl 155, K2HPO4 1.5, HEPES 10, Glucose 5, pH: 7.4), during 40 min at 37°*C*. Sensory ganglia were mechanically dissociated using a polished Pasteur pipette and neurons were plated on collagen-coated cover glasses, maintained in MEM media (Earle's salts, GIBCO-Thermo Fisher Scientific 41090036) supplemented with MEM-vit (GIBCO-Thermo Fisher Scientific 11120052), 10% FBS (Invitrogen-Thermo Fisher Scientific 16000044), 200μg/mL streptomycin, 125μg/mL penicillin, and nerve growth factor (NGF mouse 7S, 100 ng/ml; Sigma N0513). Primary sensory neurons were recorded 1 day after plating.

## 3. Results

### 3.1. Mechanical features of PC12 neurites

The geometrical features of axons and neurites are shared by primary cell cultures such as spinal, cortical, sensory, dorsal root ganglia or hippocampal neurons and by many cell lines (H19-7/IGF-IR, SK-N-BE(2), BE(2)-C, SH-SY5Y or RN33B). Among cell lines, the PC12 cells were first proposed as a research model system by Lloyd A. Greene in 1976 (Greene and Tischler, 1976). The phenotype features of the PC12 have been a key factor to be used as a neuron model (Yankner and Shooter, 1982; Guroff, 1985; Jacobs and Stevens, 1986a,b; Teng et al., 2006; Westerink and Ewing, 2008). Furthermore, in terms of mechanical response, PC12 cell line shares mechanical characteristics with primary cell cultures (Dennerll et al., 1989; Lamoureux et al., 1989; Bernal et al., 2007, 2010; Grevesse et al., 2015). Therefore, fluctuations-based measurements of neuron-like cells can determine the contribution of the axon-skeleton and plasma membrane features to cellular mechanical properties, analyzing the power spectral density (Equations 1, 2 and 3, and Figure 2C).

The obtained experimental *PSD* summarize the contribution of all mechanical features of the PC12 neurite sample (Figure 2C). For instance, at low frequencies (below 1Hz) the *PSD* is dominated by the axial tension σ of the neurite/string (Figure 3A), meaning that the membrane contribution (Equation 1) can be neglected in the total *PSD*_*n*_ (Equation 3). Therefore, in this regime the dispersion relation for the string, $\omega_{s}=\left(Bq^{4}+\sigma q^{2}\right)/\left(4\eta_{s}^{e}\right)$, can be simplified as ω_*s*_≈$\sigma q^{2}/4\eta_{s}^{e}$ and the *PSD* of the neurite is approximate to *PSD*_σ_≈$\left(k_{B}T/\sqrt{2^{6}\pi^{3}\eta_{s}^{e}\sigma }\right)f^{-3/2}$ (Figure 3). In the same way, the flexural rigidity of the neurite/string B, contributes to the *PSD* only in a range of frequencies that are determined by the reduced dispersion relation ω_*s*_≈$Bq^{4}/4\eta_{s}^{e}$, leading to an approximated solution $PSD_{B}$≈$\left(\csc\left(\pi /8\right)k_{B}T/\left[16\times 2^{3/4}\pi^{7/4}B^{1/4}\eta_{s}^{e\;3/4}\right]\right)f^{-7/4}$ dominated by B (Figure 3B). The frequency regimes can be estimated using reported values of the axial tension and the flexural rigidities of different biological samples (Table 1). The minimum and maximum *q*-vectors for the neurite geometry are determined by: $q_{s}^{\min}=\pi /L$ and $q_{s}^{\max}=q_{r}=1/r$ (Figure 1E). These *q*-vector limits leads to the frequency range shown in Figures 3A,B (dark gray area bottom panels).

**Table 1:** Parameter values available in the literature for PC12 cells, sensory neurons from mouse Dorsal Root Ganglia (DRG mouse), Hippocampal neurons from mouse (Hippo mouse), sensory neurons from chicken Dorsal Root Ganglia (DRG chick) and others.

**Symbol**	**Mechanical**	**PC12**	**DRG**	**Hippo**	**DRG**	**Other**	**Units**	**References**
	**parameter**		**mouse**	**mouse**	**chick**			
$\eta_{s}^{e}$	Effective neurite viscosity	1	−	−	−	−	Pa·s	Hill et al., 2004
σ	Neurite axial tension	~0.4	0.2−0.6	0.01−0.2	~1	−	(× 10^−9^) N	Dennerll et al., 1989; Lamoureux et al., 1989; Koch et al., 2012; Gárate et al., 2015
B	Neurite flexural stiffness	−	−	−	−	0.2−2	(× 10^−23^) N·m^2^	Gittes et al., 1993; Kikumoto et al., 2006
δ	Cortex thickness	−	−	−	−	10^2^	(× 10^−9^) m	Alert et al., 2015
$\eta_{m}^{e}$	Effective cytosol viscosity	−	−	−	0.1	(1−10) × 10^−3^	Pa·s	Fushimi and Verkman, 1991; Dai and Sheetz, 1995; Weiß et al., 2013; Bandyopadhyay et al., 2014
γ	Membrane surface tension	~10^−7^	−	−	~10^−6^	5 × 10^−5^	N/m	Dai and Sheetz, 1995; Betz et al., 2009; Alert et al., 2015; Gárate et al., 2015
K	Membrane bending stiffness	−	−	−	2.5 × 10^−1^	10^−1^	(× 10^−18^) N·m	Dai and Sheetz, 1995; Betz et al., 2009; Alert et al., 2015
ρ_*b*_	Linkers density	−	−	−	−	10^14^	1/m^2^	Alert et al., 2015
*k*	Linker spring constant	~5 × 10^−6^	−	−	~2 × 10^−5^	10^−4^	N/m	Dennerll et al., 1989; Bernal et al., 2007; Alert et al., 2015

Considering now only the membrane contribution, the total *PSD*_*n*_ (Equation 3) can be reduced solely to the *PSD*_*m*_ of the membrane (Equation 2, and dispersion relation $\omega_{m}=\left(Kq^{4}+\gamma q^{2}+\rho_{b}k\right)/\left(4\eta_{m}^{e}q\right)$). Then, following the same procedure for the string, the *PSD* for each membrane mechanical parameter can be approximated as: The regime dominated by the plasma membrane tension γ leads to $\omega_{m}\approx \gamma q/4\eta_{m}^{e}$ and $PSD_{\gamma }\approx \left(k_{B}T/4\pi \gamma \right)f^{-1}$ (Figure 3C); while the case when the plasma membrane bending stiffness K dominates results in $\omega_{m}\approx Kq^{3}/4\eta_{m}^{e}$ and its theoretical solution of the power spectrum to $PSD_{K}\approx \left(k_{B}T/\left[24\pi^{5/3}K^{1/3}\eta_{m}^{e\;2/3}\right]\right)f^{-5/3}$ (Figure 3D). The wave vector limits, associated to the membrane, are determined now by $q_{m}^{\min}=q_{r}=1/r$ and $q_{m}^{\max}=2\pi /\delta_{n}$ (Figure 1E). The results of applying those two approximations are graphically shown in Figures 3C,D (dark gray area bottom panels).

The third membrane parameter describes the mechanical coupling between the plasma membrane and axon-skeleton denoted by ρ_*b*_*k*. For a regime dominated by ρ_*b*_*k*, the dispersion relation is approximated to ω_*m*_ ≈ $\rho_{b}k/4\eta_{m}^{e}q$. However, the theoretical expression for this regime cannot be obtained due to the singularity of the resulting function. Moreover, the coupled-dominant regime is obscured by the generally stronger bending rigidity contribution at high frequencies which is described above. Nevertheless, the value of this parameter can be obtained using the plateau at the low-frequency range as shown by the green segmented fit line in the Figure 2C. Thus, due to the existence of the plateau at low frequencies (Figure 1E, bottom panel), the solution at *f* → 0 can be approximated by $PSD_{f\to 0}^{\rho_{b}k}\approx \left(4\eta_{m}^{e}k_{B}T/\left[3\pi \rho_{b}^{2}k^{2}\right]\right)\Delta q^{3}$ (Figure 3E), where $\Delta q=q_{m}^{\max}-q_{m}^{\min}$ (Alert et al., 2015).

In order to extract the numerical value for each mechanical parameter, we measure the local power-law exponent and its range of frequencies from the experimental *PSD* as shown in the bottom panels in Figure 3. Each theoretical approximation is valid for its particular frequency range. This statement is confirmed by comparing the theoretical and experimental power-law (Gárate et al., 2015). In Figure 3, we present the density distribution of all mechanical features of PC12 neurites, illustrating the approximate ranges of frequencies where the theoretical solutions are valid. The robustness of the results allows us to characterize the mechanical properties of the PC12 cell neurites.

From these probability distribution (Figure 3) we extract the average and standard deviation of the mechanical parameters considered in our model (Equations 1, 2, and 3). Using the approximated solutions that we have described, the effective neurite viscosity times the neurite axial tension is in the range of $\eta_{s}^{e}\sigma \;$ = $\;{\left(2^{6}\pi^{3}\right)}^{-1}{\left(k_{B}T\times 10^{-A_{\sigma }}\right)}^{2}=\left(16.87-43.33\right)\times 10^{-12}$(N^2^·s/m^2^), where *A*_σ_ is the mean value of the probability distribution shown in Figure 3A. For the flexural neurite stiffness we obtain $\eta_{s}^{e}B={\left(2^{3}\pi^{7}\right)}^{-1}\left(\frac{\csc\left(\pi /8\right)}{16}k_{B}T\times 10^{-A_{B}}\right)=\left(2.89-31.47\right)\times 10^{-24}\left({\text{N}}^{4}\cdot {\text{s}}^{3}/{\text{m}}^{4}\right)$. For the plasma membrane surface tension $\gamma ={\left(4\pi \right)}^{-1}\left(k_{B}T\times 10^{-A_{\gamma }}\right)=\left(2.48-3.01\right)\times 10^{-5}$(N/m). For the plasma membrane bending it reads $\eta_{m}^{e}k={\left(24\pi^{5/3}\right)}^{-3}{\left(k_{B}T\times 10^{-A_{K}}\right)}^{3}=\left(0.09-0.38\right)\times 10^{-21}\left({\text{N}}^{3}\cdot {\text{s}}^{2}/{\text{m}}^{3}\right)$. Finally, for the membrane to cytoskeleton coupling we obtain $\rho_{b}^{2}k^{2}/\eta_{m}^{e}=\left(4/3\pi \right)k_{B}T\Delta q^{3}\times 10^{-A_{\rho_{b}k}}=\left(4.96-6.21\right)\times 10^{18}$(N/m^4^·s).

Note that the only mechanical parameter obtained solely from our measurements only is the plasma membrane surface tension (via using the approximated solutions). Thus, in order to obtain the rest of the mechanical parameters, an estimation of both effective viscosities ($\eta_{s}^{e}$ and $\eta_{m}^{e}$) is required, which can be taken from the literature (Table 1).

**Figure 4:**
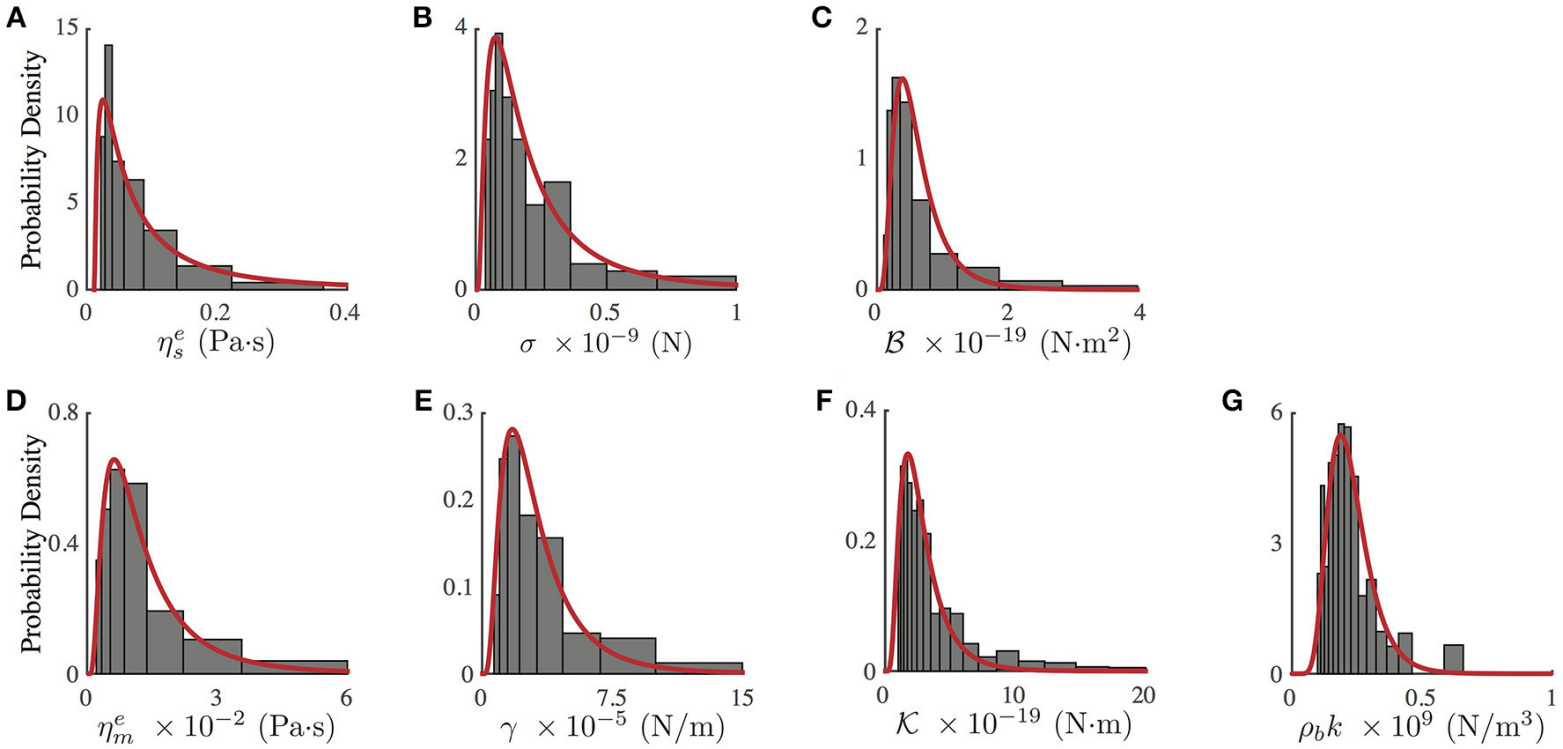
Probability density distribution of mechanical parameters of PC12 neurites, obtained from the fit of the experimental and theoretical *PSD* (Equation 3, Figure 2C red continuous line). **(A)** Effective neurite viscosity $\eta_{s}^{e}$. **(B)** Neurite axial tension σ. **(C)** Neurite flexural rigidity B. **(D)** Effective membrane viscosity $\eta_{m}^{e}$. **(E)** Membrane surface tension γ. **(F)** Membrane bending rigidity K. **(G)** Membrane to cytoskeleton coupling ρ_*b*_*k*. All experimental probability distributions were fitted by a Log-Normal distribution. *N* = 75 neurites of similar radius and length.

Notwithstanding the foregoing, it is also possible to use the full expression of the theoretical *PSD*_*n*_ (Equation 3) by a direct comparison of the experimental *PSD* (Figure 2C) to the model by a least square minimization, thus obtaining all the mechanical parameters of PC12 neurites. As shown by Betz and Sykes (Betz and Sykes, 2012), each mechanical parameter changes in a particular way the shape of the *PSD* in a given range of frequencies, thus ensuring an independent measurement of these parameters. Then, the values obtained in Figure 3 can be used as initial guesses for the mechanical parameters. Indeed, as shown in Figure 4, the parameters obtained from the numerical fit (Figure 2C, red continuous line) display well-defined probability distributions from which we can characterize the mechanical features of the PC12 neurites (Table 2).

**Table 2:** Mechanical parameter values obtained from the experimental probability distributions (Figure 4).

**Symbol**	**Mechanical parameter**	**Mode**	**Median**	**Mean**	**Units**
$\eta_{s}^{e}$	effective neurite viscosity	0.01	0.06	0.14	Pa·s
σ	neurite axial tension	0.07	0.17	0.27	(× 10^−9^) N
B	neurite flexural stiffness	0.72	0.77	0.79	(× 10^−19^) N·m^2^
$\eta_{m}^{e}$	effective cytosol viscosity	0.58	1.05	1.43	(× 10^−2^) Pa·s
γ	membrane tension	1.71	2.66	3.31	(× 10^−5^) N/m
K	membrane bending stiffness	1.72	2.42	2.87	(× 10^−19^) N·m
ρ_*b*_*k*	membrane-cytoskeleton coupling	1.89	2.16	2.30	(× 10^8^) N/m^3^

### 3.2. Axial tension and effective viscosity vs. F-actin linear density

To expand even more the versatility of the TFS technique, we couple a dichroic mirror to incorporate epifluorescence imaging to our setup (Figure 2A). Using this hybrid instrument it is now possible to study the concentration of a given protein simultaneously with the measurement of mechanical parameters of the same sample in a single apparatus.

**Figure 5:**
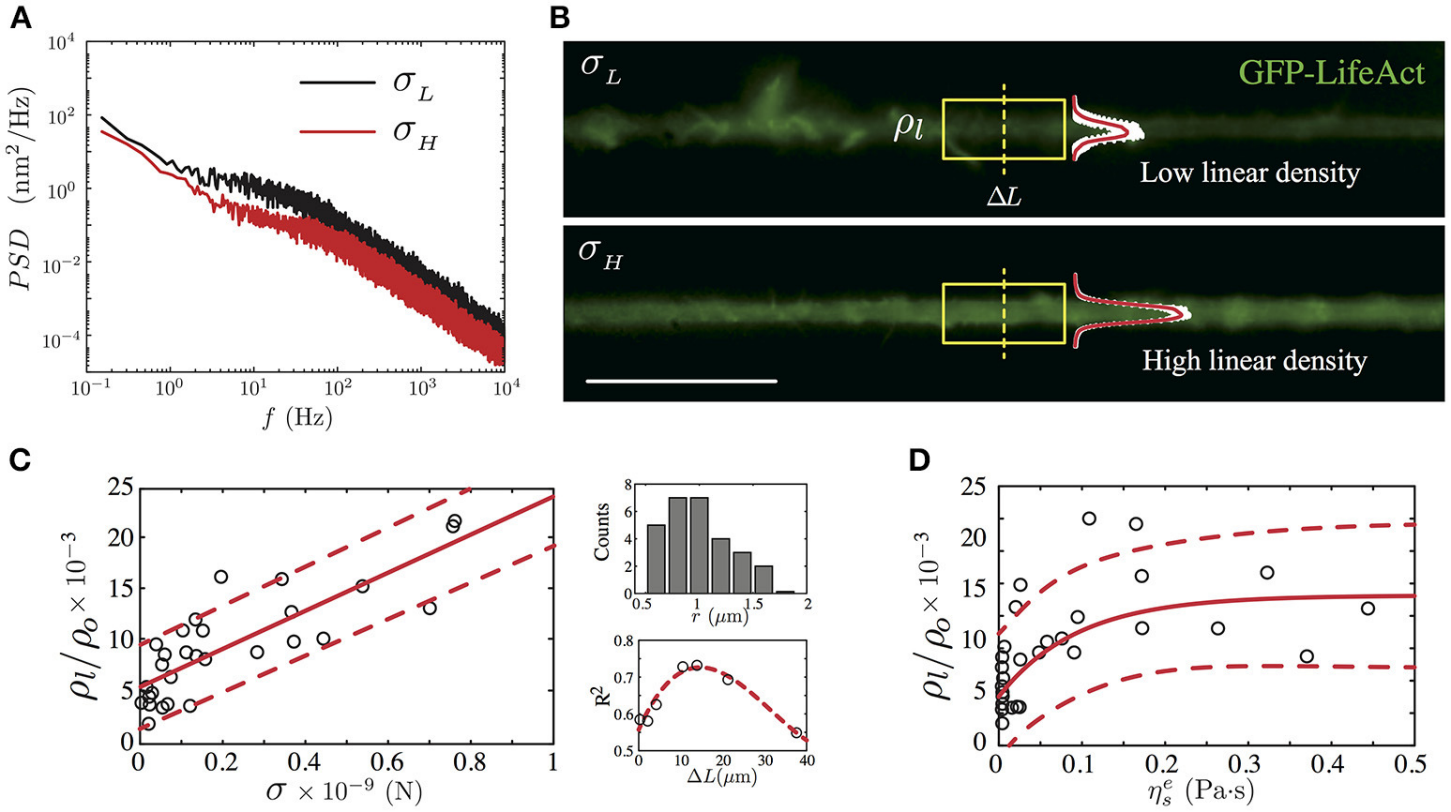
GFP-LifeAct cytoskeleton imaging of PC12 neurite samples after 4 days of growth. **(A)** Power Spectrum Density of two PC12 neurites, where the black solid line represents a neurite whose mean fluctuation amplitude is of the order of 9.47 ± 0.15 nm and an axial tension value of 0.16 ± 0.01 nN, whereas the red solid line represents a neurite with a mean fluctuation amplitude value of 7.38 ± 0.16 nm and an axial tension value of 0.45 ± 0.04 nN. **(B)** Irregular F-actin distribution in a PC12 neurite with a low actin presence $\rho_{l}/\rho_{o}=\left(4.24\pm 1.83\right)\times 10^{-3}$ (top panel). Homogeneous F-actin distribution in a PC12 neurite with a high actin presence $\rho_{l}/\rho_{o}=\left(12.76\pm 4.39\right)\times 10^{-3}$ (bottom panel). The linear density is computed using the section framed in yellow. The mean intensity profile and its standard deviation are plotted to show the intensity variations in both cases. Scale bar 20μm. **(C)** The main panel shows the linear trend between the normalized linear F-actin density (ρ_*l*_/ρ_*o*_) and the neurite axial tension (the red solid line stand for the best linear fit). Whereas the secondary panels display the neurite radii distribution and the R-square correlation value as a function of the segment Δ*L*. **(D)** The dependency of the normalized linear F-actin density as a function of the neurite effective viscosity (the red solid line stand for the best exponential fit). The red segmented line represents the confidence bound with the 95% of the data in the main plots of **(C,D)**. For the F-actin labeled version of the PC12, *N* = 28 neurites were imaged prior to the TFS measurement.

Knowing that the mechanical tension is generated and maintained by interactions between actin filaments and protein motors along the neurite shaft and at the growth cone region as well (Lamoureux et al., 1989; O'Toole and Miller, 2011; Ahmed et al., 2012; Ahmed and Saif, 2014), we focus in the next experiment on elucidating the relationship between the F-actin presence and the neurite axial tension (Figure 5A). To achieve this goal, we performed 28 experiments using F-Actin labeled PC12 neurites (Materials and Methods). The fluorescent image of the F-actin shown in Figure 5B was recorded prior to the TFS measurement (Figure 5A). From the image, we calculated the integral intensity of a 13.25μm neurite length (Figure 5B, the yellow frame corresponds to 100 pixels length), obtaining in this way the linear F-actin density, previously defined in section 2.

From our F-actin labeled PC12 neurites experiments, we have found a positive correlation between the neurite axial tension and the normalized linear F-actin density. As shown in Figure 5C, for neurites with radii within *r* = 1.18 ± 0.37 μm (Figure 5C, top right panel) the normalized linear F-actin density increases in a linear fashion with respect to the neurite axial tension with a slope equal to (18.59 ± 5.14) × 10^3^ using a neurite shaft segment Δ*L* = 13.25μm (R-square value of 0.74). This neurite segment length was chosen by comparing the R-squared values of various segment lengths (Figure 5C, top right panel). The normalized linear density vs. the axial tension slope varies in less than 10% for a segment length of the order of 2μm (R-square 0.58).

On average, the neurites with low axial tension (σ < σ_mode_, Table 2), display large spatial variations of the F-actin (Figure 5B, top panel). Thus, we define the low limit of the linear density value as $\rho_{l}^{\text{low}}={\left(\rho_{l}/\rho_{o}\right)}_{\sigma <\sigma_{\text{mode}}}=\left(5.35\pm 2.42\right)\times 10^{-3}$. In contrast, the neurites with axial tension above the median (σ > σ_median_, Table 2), show a more homogeneous intensity distribution (Figure 5B, bottom panel), which allows us to define the high limit of the linear density value as $\rho_{l}^{\text{high}}={\left(\rho_{l}/\rho_{o}\right)}_{\sigma >\sigma_{\text{median}}}=\left(12.09\pm 4.48\right)\times 10^{-3}$.

In terms of the effective viscosity of the PC12 neurite, we have found a relationship between the presence of F-actin and the viscosity due to this cytoskeletal component. As shown in Figure 5D, we observed that the neurites with low linear actin density ($\rho_{l}/\rho_{o}<\rho_{l}^{\text{low}}$) and low axial tension (σ < σ_mode_) present a mean effective viscosity equal to $\eta_{s}^{e}=\left(0.45\pm 0.24\right)\times 10^{-2}$ Pa·s which is four-fold the cytosol viscosity measured in CCL-26 epithelial cells (Bandyopadhyay et al., 2014) and mouse dendritic cells (Leduc et al., 2011). Whereas, in the case of PC12 neurite with high linear actin density ($\rho_{l}/\rho_{o}>\rho_{l}^{\text{high}}$), their mean effective viscosity is found to be $\eta_{s}^{e}=\left(0.19\pm 0.07\right)$ Pa·s which is one-fifth of the effective viscosity measured in PC12 neurites reported by Hill et al. (2004), where the high viscosity value is due to kinesin protein motors of PC12 neurites cultured up to 7 days.

No clear relationship was found when comparing the computed the F-actin linear density and the plasma membrane effective viscosity $\eta_{m}^{e}$ of this group of labeled PC12 neurites.

### 3.3. Effect of biochemical perturbations on the cytoskeleton

In this section, we study the changes in the *PSD* of PC12 neurites by; Latrunculin-A (Lat-A) and Paraformaldehyde (PFA). The purpose of these experiments is to test the sensitivity of the TFS based measurements to determine if we are able to detect the changes in the mechanical parameters caused by these two agents.

To quantify the effect of these drugs, we record the transversal amplitude fluctuations of two groups of seven wild-type PC12 neurites each. Then, one group was exposed to Lat-A at 0.2μg/mL (Sigma-Aldrich L163) for 10 min. The second group was incubated with 4% PFA for 30 min. Then, for both groups, we repeat the TFS measurements.

**Figure 6:**
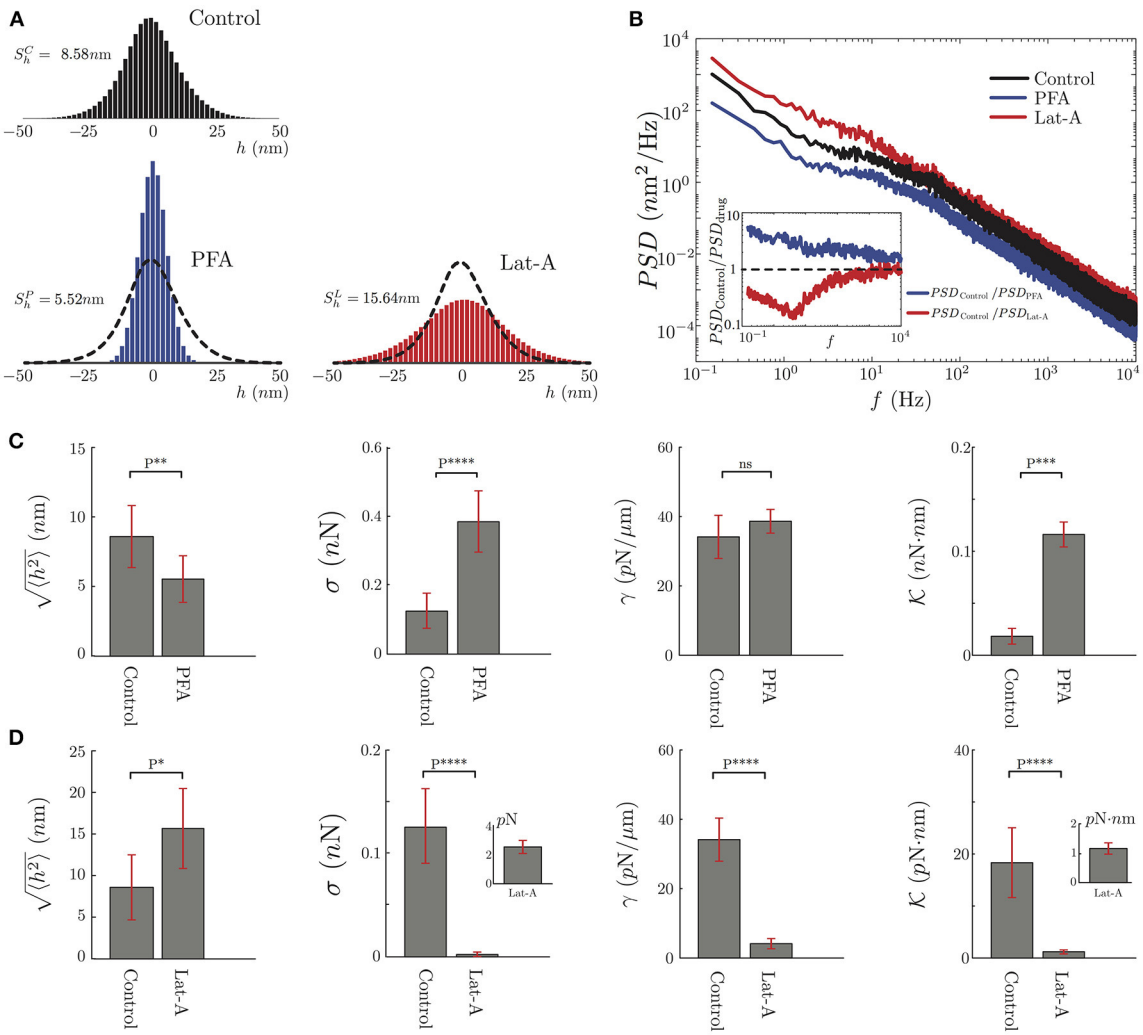
Mechanical characterization of PC12 neurites under effects of PFA and Lat-A. **(A)** The average amplitude fluctuation distribution of the seven plus seven control neurites (*N* = 14), and the same seven neurites under PFA (*N* = 7) or Lat-A (*N* = 7). **(B)** Average *PSD* of the Control, PFA and Lat-A PC12 neurites groups. The inset displays the ratio of the averaged altered *PSD* taking as a reference the average control *PSD*. **(C,D)** Quantification of the PFA and Lat-A, respectively, effects in the PC12 neurite mechanical parameters: axial tension σ, plasma membrane tension γ, plasma membrane bending rigidity K.

As seen in Figure 6, our results show a significant difference between the fluctuations of control neurites and neurites under PFA or Lat-A. For instance, the mean of the fluctuations was reduced by 35% for neurites under PFA, and was increased by more than 80% when they were exposed to Lat-A (Figure 6A). The results are consistent with the known changes in the cytoskeleton architecture provoked by these drugs. PFA causes covalent cross-links between the molecules of the cell cytoskeleton and the plasma membrane. Because of this increment of cross-linking, the cellular structure becomes more rigid, which explains the reduction of the mean of the fluctuations. A further analysis of the data shows that the effect of PFA goes beyond the reduction of the mean amplitude. The *PSD* calculation under the action PFA shows a reduction of the amplitude of the *PSD* across all frequencies (Figure 6B).

Comparing the control and PFA results, we define the ratio *PSD*_Control_/*PSD*_PFA_. We choose this definition knowing that all the mechanical parameters are located in the denominator of the approximate solutions. Thus, this ratio shows in which part of the spectrum the mechanical parameters increase or decrease. As shown in the inset of Figure 6B, the effect of the PFA, in general, increases all the mechanical parameters described in this study.

In contrast to PFA, Latrunculin-A inhibits the polymerization of the actin monomers. It also has an effect on the F-actin structures by reducing the actin filaments length, resulting in a depolymerization of the actin cytoskeleton (Yarmola et al., 2000) that can be translated in a decrease of the elasticity of the cytoskeleton. This is consistent with the increment of the mean amplitude of the fluctuations (Figure 6A). Unlike PFA, in the case of Lat-A, the *PSD* analysis shows a more localized change at low frequencies, where the axial tension is dominant in the spectrum, whereas at high frequencies the ratio between the *PSD*s is close to 1 (Figure 6B).

After this qualitative comparison, we analyze the *PSD* of the PFA, and Lat-A PC12 neurite groups to extract their mechanical parameters fitting the Equation 3 to our data. Despite the small size of the sample in each group, these results have been compared to the same neurites with and without biochemical perturbation. It follows that the variation of the mechanical parameters is not statistical but quantified for each neurite individually. As we observe in Figure 6C, PFA reduces the amplitude of the fluctuations by 35% while increasing the axial tension over 160%, the plasma membrane tension by 14% and the bending rigidity by over 400%. Whereas in Figure 6D, the effects of the Lat-A increases the amplitude of the fluctuations over 80% while reducing the axial tension, the plasma membrane tension, and the plasma membrane bending rigidity by more than 90%. Except for the plasma membrane surface tension of Control vs. PFA neurites, in all the other quantifications the results show a statistically significant change.

### 3.4. Dorsal root ganglion neurites: a more realistic case

**Figure 7:**
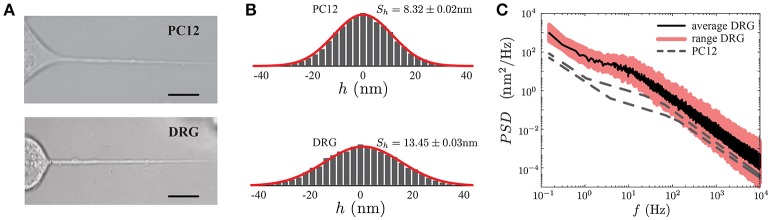
Comparison of PC12 and DRG neurites. **(A)** PC12 neurite (top) and DRG mouse neuron (bottom) of similar radii and length. Scale bars correspond to 10μm. **(B)** Probability density functions of the fluctuation amplitude for a PC12 neurite (top, *S*_*h*_ = 8.32 ± 0.02 nm) and DRG neuron (bottom, *S*_*h*_ = 13.45 ± 0.03 nm) respectively. **(C)** Power Spectrum Density of DRG neuron. The continuous black line corresponds to the average *PSD* of the DRG samples (*N* = 5), whereas the pink region stands for the minimum and maximum *PSD*'s of the collected data to show the variability range of these five DRG neurons. As a reference, the PC12 neurites *PSD* shown in Figure 5A are displayed in gray segmented lines.

Although PC12 phenotype after NGF exposure resembles sympathetic neurons, and PC12 processes have been extensively used as a model to study the mechanical properties of axons (Dennerll et al., 1989; Bernal et al., 2007, 2010; Gárate et al., 2015), we wanted to test if the general features of the *PSD* observed in PC12 neurites (Figures 3, 7A, top panel), with those obtained from actual neurons. To this end, we chose sensory Dorsal Root Ganglia (DRG) neurons (Figure 7A, bottom panel). These pseudo-unipolar neurons from the peripheral nervous system have axons but lack dendrites. The DRGs share with their counterparts of the central nervous system important features of their cytoskeleton architecture such as the actin and betaII spectrin periodicity (D'Este et al., 2015). Moreover, studies of different pathologies such as Charcot-Marie-Tooth disease or Friedreich's ataxia showed an abnormal organization of the cytoskeleton components in DRG neurons (Mollá et al., 2017; Zhao et al., 2017). In this context, real-time thermal fluctuation spectroscopy in DRG neurons provides an opportunity to assess in the future the impact of these alterations on the mechanical properties of the neurons (Perrot and Eyer, 2009; Young, 2009; Askarova et al., 2011).

Geometrically DRG neurites are similar to the PC12, both neurites types share the same length scales (neurite length *L*, the radius *r* and the plasma membrane thickness δ_*n*_). Thus, in principle, the TFS method should successfully perform a mechanical characterization of DRG. Figure 7 shows a qualitative comparison between the *PSD* of PC12 and DRG neurites with similar length and radius. Furthermore, the power spectral density in DRG neurites shows that the spectrum features are not exclusive of PC12 neurites. These results indicate that TFS technique can potentially be extended to a more ambitious scenario, as the mechanical characterization of neurons with neurodegenerative diseases.

## 4. Discussion

In this article, we have revised the Thermal Fluctuation Spectroscopy approach in combination with Optical Tweezers applied to the neurite cellular mechanics. The theoretical efforts in this area have allowed us to get access to the mechanical features of PC12 cells with a spatial resolution limited only by the laser spot size. Furthermore, we have discarded the existence of a *f*^−2/3^ power-law proposed in our previous study (Gárate et al., 2015). We have also demonstrated the non-invasive nature of the TFS technique under the experimental conditions described here. Indeed, the heating effects of the laser power increase up to two degrees the local temperature of the medium. However, to observe a difference in the mechanical parameters, due to a temperature change of two degrees, the neurite requires several minutes of continuous exposition to the laser.

Our data analysis shows consistent results using two independent methods. By using the local slopes that can be identified in the *PSD* spectrum (Figures 2C, 3), the range for each mechanical feature can be inferred from the experimental probability distributions as described by Gárate et al. (2015). In contrast, no assumptions are needed in a direct fit of the whole expression for the *PSD*_*n*_ to the experimental data (Figures 2C, 4).

### 4.1. Approximate solutions and statistical analysis

While the approximate solutions allow us to rapidly extract quantitative information of the neurite mechanics, only the plasma membrane surface tension can be extracted without knowing any of the effective viscosities (Betz et al., 2009; Alert et al., 2015; Gárate et al., 2015). Thus, in order to estimate the rest of the mechanical parameters, we proceed as follow: the effective viscosities that affect the amplitude of the fluctuations are given by $\eta_{s}^{e}=1/2\left(\eta +\eta_{cytoskeleton}\right)$ and $\eta_{m}^{e}=1/2\left(\eta +\eta_{cytosol}\right)$, with η as the dynamic viscosity of the fluid media, η ≈ 10^−3^ Pa·s. For the cytoskeletal neurite viscosity of PC12 neurites (Hill et al., 2004), it has been found a value of η_*cytoskeleton*_ = 1.1 Pa·s leading to effective neurite viscosity of the order of $\eta_{s}^{e}\approx 0.5$ Pa·s for neurites radius of 0.20–0.35 μm. While for the plasma membrane, the effective viscosity (Weiß et al., 2013) is of the order of $\eta_{m}^{e}\approx 2\times 10^{-2}$ Pa·s. With these viscosity values at hand, we can extract the mean axial neurite tension within the range σ = (0.03−0.08) × 10^−9^ N. We notice that this range of neurite axial tensions is extremely low. This can be explained by the viscosity value that we have used, which is related to the presence of kinesin motors and not to the cytosol viscosity (Hill et al., 2004). The neurite flexural rigidity is perhaps the mechanical feature, under this methodology, that is off the reported range or inferred values $B=\left(0.02-0.2\right)\times 10^{-19}$ N·m^2^. As shown in Figure 2C, the response of the plasma membrane is observed in almost four decades in the Fourier space which allows a better measurement of its mechanical features than the previous results shown in reference (Gárate et al., 2015). Thus, the inferred plasma membrane bending constant is in the range of $K=\left(2.18-9.40\right)\times 10^{-19}$ N·m, and the cytoskeleton to plasma membrane coupling is in the range of $\rho_{b}k=\left(3.15-3.64\right)\times 10^{8}$ N/m^3^. With this coupling value and the linker neurite spring constant *k* = 5 × 10^−5^ N/m reported in Dennerll et al. (1989) and Bernal et al. (2010), we can estimate the number of coupling elements as $N\approx \rho_{b}\varpi \pi r=50\pm 4$ protein complexes that are in direct interaction with the laser (ϖ is the laser spot size, and π*r* is the half-perimeter of the neurite).

### 4.2. Full model and least square minimization

The mean values of the mechanical parameters determined by the approximation method are used as input information to perform the least square minimization process between the experimental *PSD* (Figure 2C) and the string-membrane model (Equation 3).

For instance, in the case of the PC12 neurite, the axial tension σ is approximately one-tenth of the value found in DRG chick sensory neurons (Dennerll et al., 1989; Lamoureux et al., 1989), and is comparable to DRG mouse or Hippocampal mouse neurons (Koch et al., 2012). Also, the effective viscosity $\eta_{s}^{e}$ is approximately one-tenth of the inner viscosity inferred from the vesicle movement in PC12 neurites, which is driven by kinesin protein motors (Hill et al., 2004), rather than the cytosol viscosity (Bandyopadhyay et al., 2014). Indeed, the analysis of the whole *PSD*, using the least square minimization, delivers the true mechanical features for each neurite, summarized in the Table 2, which are distributed within the range of values accepted in the literature (Figure 4). Among the parameter values extracted from this analysis, our results for the PC12 neurite coupling constant ρ_*b*_*k* allow us to reduce the estimate for the number of coupling elements to N=33±1.

In the context of the recent advances on the neurite cytoskeleton architecture, using super-resolution microscopy (Xu et al., 2013; Zhong et al., 2014; Qu et al., 2017), the existence of actin rings structures from the soma to the growth cone in neurons with a periodicity of approximately 190 nm has been demonstrated. Nonetheless, and despite the lack of experimental evidence of the presence of actin rings in PC12 neurites, we could assume that such actin structures are a general feature of neuron cells. Then we estimate the number of coupling element per ring in a neurite as follow: the number of F-actin segments interacting with actin-binding proteins (ABP) should depend on the perimeter of the neurite. Then, for a neurite of radius *r*, the number of segments in a single ring should be of the order $N_{ring}\sim 2\pi r/l$, where *l* is the typical length of the actin segment. This length scale has not yet been reported. However, we can estimate it to be of the order of *l* ≈ 100nm. This value is based on the typical 6.7nm size of a G-Actin monomer (Kabsch et al., 1990). Given that a stable single F-Actin is typically composed of 13 actin monomers (Holmes et al., 1990), we determine that *l* is of the order of 87.1nm. This value does not consider the ABP Adducin protein that is a part of the actin ring, which could close the gap to reach 100 nm segment length. Thus, considering PC12 neurites of radius between 0.4 and 1.0μm gives us a number of $N_{ring}\approx \left(25-62\right)$ actin segments present in each ring that are able to couple the cytoskeleton to the plasma membrane.

### 4.3. Linear F-actin density

The correlation analysis between the actin fluorescent imaging and the fluctuation measurements has revealed a link between the axial tension and the effective viscosity in the PC12 neurites. First, the F-actin linear density shows a positive trend with respect to the axial tension (Figure 5C). Second, the F-actin linear density reaches a plateau for effective viscosity values above $\eta_{s}^{e}>\left(0.11\pm 0.07\right)$ Pa·s (Figure 5D).

These two results not only depend on the F-actin presence alone. Indeed, the axial tension in neurites is controlled by the activity of protein motors and also by the axonal microtubules. Nonetheless, based on the their F-actin linear density, neurites are categorized as having low or high linear densities, $\rho_{l}^{\text{low}}$ and $\rho_{l}^{\text{high}}$. Then, we are able to identify neurites with axial tension below the mode (with inhomogeneous F-actin distribution) or above the median (with homogeneous F-actin distribution) of the probability distribution of the axial tension shown in the Figure 4.

Classifying PC12 neurites by these criteria, we identify two groups of neurites: low F-actin density and low axial tension, and high F-actin density and high axial tension. The analysis of the effective viscosity under these new criteria shows that the average viscosity of the first group is comparable to the cytosol viscosity (Bandyopadhyay et al., 2014). In contrast, the analysis of the second group reveals that its effective viscosity is close to the viscosity due to the presence of kinesin motors (Hill et al., 2004). Then, within the 75 wild-types and the 28 F-actin label PC12 neurites, we have neurites at different stages of development (reminding that all the experiments were performed after 4 days of growth). Thus, the different stages of development of the PC12 neurites could explain the skewness of the probability distribution of the mechanical parameters shown in Figure 4.

### 4.4. Effects of PFA and lat-A

It is well accepted that the mechanical features of cells rely on the cytoskeletal and plasma membrane structures. Our results on the effects of Paraformaldehyde and Latrunculin-A show unmistakably that TFS based measurements in neurites provide enough sensitivity to account for the mechanical changes under biochemical perturbations (Figure 6). Indeed, while the PFA increases the cross-linking among all proteins in a cell, Lat-A disrupt and depolymerize actin structures, which decouples the cytoskeleton from the plasma membrane. In these cases, we observe an increment or decrement in the mechanical features respectively, which is coherent with the effect of each drug. This result opens a possibility to study, in long-term experiments, the temporal evolution of the mechanical features as a function of time. However, it becomes difficult to handle the thermal drift which is important for precise nanometric fluctuation measurements in such experiments.

Finally, as the *PSD* features is not a particular case of the PC12 neurites, but a more general characteristic of neuron-like cells, such as DRG neurons (Figure 7), we could now think about assessing more complex scenarios, as in cases of nervous degenerative diseases.

## 5. Conclusion

The TFS based measurements can be implemented in various ways. Here, a traditional optical tweezers setup has provided us with relevant and accurate mechanical information about mechanics of PC12 neurites. Furthermore, a simple combination of TFS with Fluorescence Imaging allows us to correlate qualitative and quantitative information that can help us to understand the role of cytoskeletal components in the neurite and their close relation to cellular mechanics.

## 6. Ethics statement

This study was performed using young adult P21 C57BL6 mice. All experiments were conducted according to bioethical guidelines of the Comisión Nacional de investigación Científica y Tecnológica de Chile (CONICYT) and have been approved by the Bioethical Committee of the University of Santiago de Chile (Reference Number 630).

## Author contributions

RB and FG conceive the experiments and build the setup. YA prepare the samples. FG and MP prepare the samples and perform the experiments. RB and FG analyzed the data and RB wrote the manuscript. RB, FG, and MP review the article.

### Conflict of interest statement

The authors declare that the research was conducted in the absence of any commercial or financial relationships that could be construed as a potential conflict of interest.
